# Genetic polymorphisms of epidermal growth factor in relation to risk of hepatocellular carcinoma: two case-control studies

**DOI:** 10.1186/1471-230X-13-32

**Published:** 2013-02-18

**Authors:** Jian-Min Yuan, Yunhua Fan, Simona Ognjanovic, Renwei Wang, David Van Den Berg, Sugantha Govindarajan, Mimi C Yu

**Affiliations:** 1 Division of Cancer Control and Population Sciences, University of Pittsburgh Cancer Institute, UPMC Cancer Pavilion, Suite 4C, 5150 Centre Avenue, Pittsburgh, PA, 15232, USA; 2 Department of Epidemiology, University of Pittsburgh Graduate School of Public Health, A529 Crabtree Hall, 130 DeSoto Street, Pittsburgh, PA, 15261, USA; 3 Masonic Cancer Center, University of Minnesota, 554 CCRB, 425 East River Road, Minneapolis, MN, 55455, USA; 4 Masonic Cancer Center, University of Minnesota, 1-185 Moos Tower, 515 Delaware Street SE, Minneapolis, MN, 55455, USA; 5 The USC/Norris Comprehensive Cancer Center, Keck School of Medicine, University of Southern California, 1441 Eastlake Avenue, Los Angeles, CA, 90089, USA; 6 Department of Pathology, Rancho Los Amingo Medical Center, 7601 E. Imperial Highway, JPI Building Basement, Downey, CA, 90242, USA

**Keywords:** Epidermal growth factor, T-helper, Cytokines, Hepatocellular carcinoma

## Abstract

**Background:**

Earlier, we reported a highly statistically significant association between T-helper 1 (Th1) and Th2 cytokine genotypes and hepatocellular carcinoma (HCC) risk among natives of southern Guangxi, China, a hyperendemic region for HCC. Epidermal growth factor (EGF) plays a critical role in malignant transformation of hepatocytes and tumor progression. A polymorphism in the *EGF* gene (61A > G) results in elevation of EGF in liver tissues and blood. Epidemiological data are sparse on the possible association between *EGF* genetic polymorphism and HCC risk.

**Methods:**

The *EGF 61A > G* polymorphism, multiple Th1 and Th2 genotypes, and environmental risk factors for HCC were determined on 117 HCC cases and 225 healthy control subjects among non-Asians of Los Angeles County, California, a low-risk population for HCC, and 250 HCC cases and 245 controls of southern Guangxi, China.

**Results:**

Following adjustment for all known or suspected HCC risk factors, non-Asians in Los Angeles who possessed at least one copy of the high activity 61*G allele of the *EGF* gene showed a statistically non-significant, 78% increased risk of HCC compared with those possessing the *EGF* A/A genotype. This *EGF-*HCC risk association significantly strengthened among heavy users of alcohol [odds ratio (OR) = 3.44, 95% confidence interval (CI) = 0.93–12.76, P = 0.065)], and among individuals carrying the high-risk Th1/Th2 genotypes for HCC (OR = 3.34, 95% CI = 1.24-9.03, P = 0.017). No association between *EGF* genotype and HCC risk was observed among Chinese in southern Guangxi, China.

**Conclusion:**

Genetic polymorphism in the *EGF* gene resulting in elevated level of EGF, may contribute to HCC risk among low-risk non-Asians in Los Angeles.

## Background

Hepatocellular carcinoma (HCC) is the sixth most common cancer worldwide and the third leading cause of cancer deaths [1]. The incidence of HCC and the distribution of HCC risk factors vary widely in different geographic regions worldwide. China and Africa are areas of high HCC incidence where the primary cause of HCC is chronic infections with the hepatitis B virus (HBV), and dietary aflatoxin exposure being an important cofactor [2,3]. In low HCC incidence areas including Europe and North America, diverse environmental factors, including chronic HBV and hepatitis C virus (HCV) infections, heavy alcohol use, diabetes, obesity, and tobacco use have been shown to contribute to the local burden of HCC [3-6]. However, only a minority of people with established risk factors eventually develop HCC, suggesting that other environmental and/or genetic factors may play a role in HCC development.

Among currently well-established risk factors, inflammation represents a common molecular pathway in hepatocarcinogenesis [7]. Acute inflammation develops in response to infectious agents or tissue injury where initially pro-inflammatory cytokines (Th1) are released to fight off infection or induce tissue remodeling. Once the infection is cleared or tissue damage is healed, the anti-inflammatory (Th2) cytokines are released to resolve inflammation and establish homeostasis. In chronic inflammation, however, the inflammatory process persists over prolonged periods of time and the continual cell death/regeneration accompanying the process is recognized to enhance risk of cancer, including HCC (reviewed in [8]). In HCC, hepatic injury can be induced by viruses (HBV or HCV), alcohol, or aflatoxin exposure [7]. The resultant necro-inflammatory process, where necrosis is followed by hepatocyte proliferation, leads to a continuous cycle of cell destruction/regeneration characterized by abnormal nodules surrounded by collagen deposit and tissue scarring. The nodules can progress from a hyperplastic to a dysplastic phase and ultimately to HCC [7].

Epidermal growth factor (EGF) is a mitogen for hepatocytes [9], and plays a critical role in liver tissue regeneration [10]. Mounting evidence supports a role for EGF in malignant transformation, tumor growth and progression [11]. Over-expression of a secreted human EGF fusion protein enhances the transformation of fibroblasts to fibrosarcomas and induces the development of HCC in transgenic mice [12,13]. Gene expression profiles comparing normal liver tissue with liver tumors in these mice suggest a role for an autocrine mechanism during EGF-induced hepatocarcinogenesis [12]. A functional polymorphism in *EGF* at position 61 (A > G) (SNP rs4444903) was recently identified with the *G/G* genotype associating with higher gene expression compared to the *A/A* genotype [14]. Similarly, increased EGF expression was reported in serum and liver tissue from HCC patients with the *G/G* genotype [14], and cirrhotic patients with the *G/G* genotype were more likely to progress and develop HCC than cirrhotic patients with the *A/A* genotype [15]. Interestingly, *G/G* genotype was associated with elevated risk of other malignancies, including glioma [16], malignant melanoma [14], gastric cancer [17], esophageal adenocarcinoma [18], and lung cancer [19].

Utilizing two existing case–control study databases, we examined the association between *EGF 61A > G* polymorphism and HCC risk in two populations at polar ends of the HCC risk spectrum. The Chinese in southern Guangxi, China, exhibit the highest recorded incidence rate of HCC in the world (120 per 100,000 person-years in men) [20] while the non-Asians in Los Angeles, California, USA possess one of the lowest incidence rate of HCC in the world (4 per 100,000 person-years among non-Hispanic white men) [21]. In the present study, we also examined the *EGF* genotype – HCC risk association across different risk strata defined by genetic profiles (Th1/Th2 cytokine genotypes) or environmental exposures (use of alcohol or tobacco, hepatitis serology status) within each of the two study populations.

## Methods

The present study included participants of two case–control studies of HCC, one conducted among low-risk non-Asians in Los Angeles, California, and the other conducted in high-risk Chinese in the southern part of the Guangxi Autonomous Region, China. The designs of the two studies have been described previously [6,22]. Permission to conduct this study had been obtained from the Institutional Review Boards at the University of Southern California and the Guangxi Cancer Institute. Separate informed consent forms for interview and biospecimen collection were obtained from each study participant.

### HCC patients

In Los Angeles, we studied incident HCC in black, and Hispanic and non-Hispanic white residents of Los Angeles County, who were between 18 and 74 years of age at diagnosis from January 1984 through December 2001. Cases were identified through the Los Angeles County Cancer Surveillance Program, a population-based cancer registry that records all incident cancers diagnosed in residents of Los Angeles County. Due to the rapidly fatal nature of HCC (the median time interval between diagnosis and death is approximately 3 months), 84% of eligible patients died prior to our attempted contact. Among the 478 patients we contacted, 34 (7%) were too ill to be interviewed, and 325 (73%) of the remaining 444 were interviewed. An experienced hepatopathologist reviewed the histology slides of all interviewed HCC patients; 25 cases judged to be non-HCC were excluded. Virtually all histologically confirmed HCC patients had an underlying cirrhosis.

In Guangxi, China, we identified newly diagnosed HCC from four major hospitals in the city of Nanning. Participating hospitals were comparable in their quality of patient care and diagnosis. Only patients diagnosed during September 1995 through September 1998, between the ages of 20 and 64 years, and residing in Nanning City or its neighboring townships were asked to participate in the study. We began the study in October 1995 and closed enrollment in October 1998 when 250 patients had been recruited into the study. Among the 250 HCC patients, 40 (16%) were diagnosed histologically, 162 (65%) were diagnosed by positive serum α-fetoprotein level above 500 ng/ml persistent for more than one month together with supportive imaging/clinical evidence, and 48 (19%) were diagnosed with imaging/clinical evidence only.

### Control subjects

In Los Angeles, we sought to recruit up to two control subjects per case from the neighborhoods where HCC patients resided at the time of diagnosis, who were matched to the index case by sex, age (within 5 years), and race (Hispanic white, non-Hispanic white, black). A total of 474 neighborhood control subjects were recruited into the study; most were the first (74%) or second (12%) eligible neighbors.

In Guangxi, China, we identified one consenting control subject per case among all patients admitted to the same hospital within one month of the index case’s hospital admission, who had no history of cancer or clinical liver cirrhosis. The matching criteria were age (within 3 years), gender, ethnicity (Han, Zhuang, Yao, other), and district (if resident of Nanning City) or township (if resident of neighboring townships) of residence.

### Data collection

All consenting cases and control subjects in Los Angeles, California and Guangxi, China were interviewed in person by trained interviewers using structured questionnaires. Both the Los Angeles and Guangxi questionnaires solicited demographic information, lifetime use of tobacco and alcohol, medical history, and other lifestyle factors. An alcohol drinker was defined as someone who had drunk alcoholic beverages at least once a week for six months or longer. One drink was defined as 360 g of beer (12.6 g of ethanol), 103 g of wine (12.3 g of ethanol), or 30 g of spirit (12.9 g of ethanol). A smoker was defined as someone who had ever smoked on a daily basis. Smokers were asked at what age (years) they began smoking on a daily basis, the average number of cigarettes smoked per day, and total number of years of smoking. Former smokers were asked about the number of years since smoking cessation.

Serum and buffy coat samples were collected from all subjects of the Guangxi study (250 cases and 250 controls). For the Los Angeles study, we collected from study subjects serum samples beginning in January 1992 and buffy coat samples beginning in October 1995. The buffy coat samples were available on 120 (73%) of 164 eligible HCC cases (i.e., those interviewed after October 1995). For the 277 control subjects from whom DNA donation was sought, 230 (83%) consented and donated blood samples. We examined and found no differences in the distributions by age, gender, level of education, cigarette smoking, alcohol consumption, history of diabetes, and serologic markers for HBV and HCV infections between subjects with DNA (i.e., those included in the present study) and those without DNA, both for the HCC cases and for the control subjects.

### Laboratory tests

Blood samples from cases and controls were processed and stored (-20°C) in an identical manner. The assays used for testing serologic markers of HBV and HCV infections have been described previously [6,22]. Briefly, we tested all study samples for the presence of hepatitis B surface antigen (HBsAg) in serum using commercialized kits (AUSRIA, Abbott Laboratories, North Chicago, IL), and negative samples (for the Los Angeles study only) were further tested for the presence of antibodies to the hepatitis B core antigen (anti-HBc) using standard testing kits (Corab, Abbott Laboratories, North Chicago, IL). All samples were tested for the presence of antibodies to the hepatitis C virus (anti-HCV) in serum using the ELISA version 2.0 kit manufactured by Ortho Diagnostic Systems, with confirmation of positive samples using RIBA version 2.0 (Chiron, Emeryville, CA). Serum samples were tested blindly, identified only by codes without regard to case/control status.

DNA was purified from buffy coats of peripheral blood using a QIAamp 96 Blood Kit (Qiagen, Valencia, CA). Genotyping assay was developed for the *EGF 61A > G* polymorphisms using the fluorogenic 5'-nuclease assay (TaqMan Assay) according to the allele-specific primers described previously [15]. The genotyping assays were carried out on TaqMan using a PCR Core Reagent kit (Applied Biosystems, Foster City, CA) according to manufacturer’s instructions. The following oligonucleotide primer sequences (Integrated DNA Technology, Coralville, IA) were used for PCR amplification of cDNA, *EGF* forward: CTTGTCATGCTGCTCCTCCT, reverse: GAGGGCATATGAAAGCTTCG and β2-microglobulin forward: TTTCATCCATCCGACATTGA, reverse: ATCTTCAAACCTCCATGATG. PCR amplification using ~10 ng of genomic DNA was performed in a thermal cycler (MWG Biotech, High Point, NC) with an initial step of 95°C for 10 minutes, followed by 50 cycles of 95°C for 25 seconds and 1 minute at the annealing temperature (Ta, Appendix A). The fluorescence profile of each well was measured in an ABI 7900HT Sequence Detection System and the results analyzed with Sequence Detection Software (Applied Biosystems). Experimental samples were compared to 12 standard controls to identify the 3 genotypes at each locus. Any samples that were outside the parameters defined by the controls were identified as non-informative and were retested. We previously described in detail the assays for genetic polymorphisms in Th1 cytokine genes including interferon γ, interleukin 2 (*IL2*), *IL12 and IL18* and in Th2 cytokine genes including *IL4* and *IL10*[22,23].

Three HCC cases (all non-Asians in Los Angeles) and ten controls (5 of non-Asians in Los Angeles and 5 of Chinese in Guangxi, China) were non-informative in the *EGF* genetic polymorphism. These subjects were excluded. Thus, the present analysis included 367 HCC cases (117 non-Asians and 250 Chinese) and 470 control subjects (225 non-Asians and 245 Chinese).

### Statistical analysis

Chi-square test was used to examine differences in the distributions of selected demographic variables and the *EGF 61A > G* genotype frequencies between cases and controls by race/ethnicity. The student *t* test was used to examine the difference in age between cases and controls. Unconditional logistic regression models [24] were used to examine the associations between the *EGF 61A > G* polymorphism and risk of HCC. The strength of a gene-HCC risk association was measured by its odds ratio (OR), and its corresponding 95% confidence interval (CI) and two-sided *p* value. Given the difference in risk profiles and frequencies of *EGF A61G* polymorphism between non-Asians in Los Angeles and Chinese in Guangxi, we analyzed and presented the results separately for the two populations. Subjects’ age, sex, and race/ethnicity were included as covariates in all logistic regression models. When examining the independent effect of the *EGF 61A > G* polymorphism on HCC risk, we included additional risk factors for HCC in the logistic regression models: level of education, smoking, alcohol consumption, and serology of HBV and HCV infections.

Statistical analysis was conducted using the SAS software Version 9.1 (SAS Institute, Cary, NC). All *P* values quoted are two-sided. Two-sided *P* values that are 0.05 or less were considered statistically significant.

## Results

The mean ages (±standard deviation) of HCC patients and control subjects in Los Angeles, California, were 60.4 (±10.2) years and 59.3 (±10.7) years, respectively. The corresponding figures in Guangxi, China were 49.3 (±9.6) and 49.5 (±10.3) years. In Los Angeles, there were higher proportions of Hispanics and African-Americans in cases than controls. HCC patients attained a lower level of education than control subjects in the Los Angeles study population, but there was no difference in level of education between cases and control in the Guangxi study population. The prevalence of cigarette smoking, heavy use of alcohol, and positivity in hepatitis B and/or C serology were significantly higher in HCC patients than control subjects in both populations (Table 1).

**Table 1:** Distributions of demographic characteristics in hepatocellular carcinoma (HCC) patients and control subjects by study location

	**Non-Asians in Los Angeles, California**		**Chinese in southern Guangxi, China**	
**Demographics**	**HCC patients (n = 117)**	**Control subjects (n = 225)**	**HCC patients (n = 250)**	**Control subjects (n = 245)**
Age (years)	60.4	59.3	49.3	49.5
*2-sided P*	*0.37*		*0.87*	
Sex (%)				
Males	80 (68.4)	136 (60.4)	220 (88.0)	216 (88.2)
Females	37 (31.6)	89 (39.6)	30 (12.0)	29 (11.8)
*2-sided P*	*0.15*		*0.96*	
Race/ethnicity (%)				
Non-Hispanic white Americans	69 (59.0)	179 (79.6)	…	…
Hispanic Americans	39 (33.3)	34 (15.1)	…	…
African Americans	9 (7.7)	12 (5.3)	…	…
Chinese – Han ethnicity	…	…	198 (79.2)	196 (80.0)
Chinese – Zhuang/Yao ethnicity	…	…	52 (20.8)	49 (20.0)
*2-sided P*	*0.0002*		*0.83*	
Level of education (%)				
Below high school	22 (18.8)	17 (7.6)	115 (46.0)	102 (41.6)
High School graduates	32 (27.3)	51 (22.7)	79 (31.6)	87 (35.5)
Some college/occupational school	40 (34.2)	75 (33.3)	39 (15.6)	33 (13.5)
College graduates or above	23 (19.7)	82 (36.4)	17 (6.8)	23 (9.4)
*2-sided P*	*0.001*		*0.47*	
Cigarette smoking				
Non- or long ex-smokers	70 (59.8)	162 (72.0)	141 (56.4)	161 (65.7)
Current or recent ex-smokers	47 (40.2)	63 (28.0)	109 (43.6)	84 (34.3)
<20 cigarettes/day	14 (12.0)	26 (11.6)	33 (13.2)	28 (11.4)
≥20 cigarettes/day	33 (28.2)	37 (16.4)	76 (30.4)	56 (22.9)
*2-sided*	*0.03*		*0.09*	
No. alcoholic drinks/day				
Nondrinkers	35 (29.9)	74 (32.9)	158 (63.2)	205 (83.7)
<3	28 (23.9)	110 (48.9)	60 (24.0)	31 (12.7)
≥3	54 (46.2)	41 (18.2)	32 (12.8)	9 (3.7)
*2-sided P*	*<0.0001*		*<0.0001*	
Hepatitis B serology				
Negative	84 (71.8)	198 (88.0)	45 (18.0)	212 (86.5)
Anti-HBc positive only	30 (25.6)	27 (12.0)	…	…
HBsAg positive	3 (2.6)	0	205 (82.0)	33 (13.5)
*2-sided P*	*0.0002*		*<0.0001*	
Hepatitis C serology				
Anti-HCV negative	59 (50.4)	224 (99.6)	241 (96.4)	242 (98.8)
Anti-HCV positive	58 (49.6)	1 (0.4)	9 (3.6)	3 (1.2)
*2-sided P*	*<0.0001*		*0.09*	
Hepatitis B/C serology				
Both negative	54 (46.2)	198 (88.0)	45 (18.0)	210 (85.7)
Either positive†	63 (53.9)	27 (12.0)	205 (82.0)	35 (14.3)
*2-sided P*	*<0.0001*		*<0.0001*	

* Anti-HBC, antibodies to hepatitis B core antigen; HBsAg, hepatitis B surface antigen; anti-HCV, antibodies to hepatitis C virus.

† Positive for hepatitis B surface antigen, antibodies to the hepatitis B surface antigen (for Los Angeles subjects only), or antibodies to hepatitis C virus.

Table 2 shows the genotypic and allelic frequencies of the *EGF 61A > G* polymorphism by race/ethnicity among controls. There were no statistically significant differences in genotypic or allelic frequencies between Han and Zhuang/Yao Chinese in Guangxi, China whereas the difference in genotypic and allelic frequencies between non-Hispanic whites and Hispanics/blacks in Los Angeles, California, was statistically significant (*P* = 0.0003). The *G* allele frequencies of the *EGF 61A > G* polymorphism in non-Asians and Chinese were 0.51 and 0.30, respectively (2-sided *P < 0.001)*. For both the Guangxi Chinese and the Los Angeles non-Asians, their respective distribution of the *EGF 61A > G* polymorphism was statistically compatible with the Hardy-Weinberg equilibrium (Table 2).

**Table 2:** **The genotypic and allelic frequencies of the *EGF *polymorphism among control subjects by race/ethnicity**

**Genetic polymorphism**	**Non-Asians in Los Angeles, California**		**Chinese in southern Guangxi, China**	
	**Non-Hispanic whites (n = 179)**	**Blacks/Hispanics (n = 46)**	**Han Chinese (n = 196)**	**Zhuang/Yao Chinese (n = 49)**
***EGF 61A > G***				
*A/A*	0.21	0.50	0.48	0.49
*A/G*	0.48	0.33	0.43	0.45
*G/G*	0.31	0.17	0.09	0.06
*A*	0.45	0.66	0.70	0.71
*G*	0.55	0.34	0.30	0.29
2-sided *P for HW†*	*0.81*	*0.07*	*0.72*	*0.48*
*2-sided P‡*	*0.0003*		*0.84*	

* Test for differences in genotypic frequencies between the four racial/ethnic groups.

† Test for the Hardy-Weinberg linkage equilibrium within each racial/ethnic group.

‡ Test for differences in genotypic frequencies between the two racial/ethnic groups within a given study location.

Among non-Asians in Los Angeles, both the 61 *G/G* and the *G/A* genotypes of the *EGF* gene were associated with an increased risk of HCC. There was no dose–response relationship between the number of the 61*G alleles and HCC risk. Compared with the *A/A* genotype, individuals possessing the *A/G* or *G/G* genotypes had a borderline statistically significant 78% increased risk of developing HCC after adjustment for multiple risk factors for HCC (Table 3). Among Chinese of Southern Guangxi, no association between the *EGF* 61A > G polymorphism and HCC risk was noted (Table 3).

**Table 3:** **Comparison of *EGF *genotype frequencies in HCC patients with control subjects among non-Asians in Los Angeles, California, and Chinese in southern Guangxi, China**

**Subgroup**	***EGF 61A > G***	**Non-Asians in Los Angeles**			**Chinese in southern Guangxi**		
		Ca/Co*	**OR (95% CI)†**	**OR (95% CI)‡**	Ca/Co*	**OR (95% CI)†**	**OR (95% CI)‡**
All subjects	*A/A*	28/60	1.00	1.00	126/118	1.00	1.00
	*A/G*	61/102	1.61 (0.89–2.90)	2.04 (1.03–4.07)	99/107	0.87 (0.60–1.26)	0.69 (0.41–1.18)
	*G/G*	28/63	1.34 (0.68–2.64)	1.39 (0.64–3.01)	25/20	1.17 (0.62–2.22)	0.97 (0.38–2.46)
	*A/G + G/G*	89/165	1.52 (0.87–2.65)	1.78 (0.93–3.38)	124/127	0.91 (0.64–1.30)	0.73 (0.44–1.21)
Seronegative for all viral hepatitis markers	*A/A*	12/52	1.00	1.00	26/101	1.00	1.00
	*A/G + G/G*	42/146	1.65 (0.76–3.55)	1.95 (0.86–4.41)	19/109	0.65 (0.33–1.27)	0.61 (0.30–1.23)
Seropositive for any viral hepatitis markers^§^	*A/A*	16/8	1.00	1.00	100/17	1.00	1.00
	*A/G + G/G*	47/19	1.43 (0.50–4.07)	1.62 (0.50–5.27)	105/18	0.91 (0.44–1.88)	0.88 (0.41–1.87)
Non-drinkers or <3 drinks of alcoholic beverages per day	*A/A*	16/45	1.00	1.00	114/115	1.00	1.00
	*A/G + G/G*	47/139	1.12 (0.56–2.23)	1.60 (0.72–3.52)	104/121	0.86 (0.60–1.25)	0.75 (0.45–1.26)
3+ drinks of alcoholic beverages per day	*A/A*	12/15	1.00	1.00	12/3	1.00	1.00
	*A/G + G/G*	42/26	4.34 (1.37–13.74)	3.44 (0.93–12.76)	20/6	0.74 (0.12–4.70)	0.32 (0.02–4.83)
Non-smokers or long-term ex-smokers^¶^	*A/A*	17/40	1.00	1.00	71/78	1.00	1.00
	*A/G + G/G*	53/122	1.55 (0.75–3.21)	1.86 (0.83–4.18)	70/83	0.92 (0.58–1.45)	0.83 (0.44–1.57)
Current smokers or recent ex-smokers^¶^	*A/A*	11/20	1.00	1.00	55/40	1.00	1.00
	*A/G + G/G*	36/43	1.59 (0.63–3.99)	1.52 (0.50–4.63)	54/44	0.87 (0.49–1.54)	0.51 (0.21–1.23)

* Number of cases/number of controls.

† Adjusted for age, sex, and race; OR, odds ratio; CI, confidence interval.

‡ Further adjusted for level of education, smoking (non-smokers or long-term ex-smokers, current smokers or recent ex-smokers with <20 cigarettes/day, current smokers or recent ex-smokers with 20+ cigarettes/day), alcohol consumption (non-drinkers, <7, 7–14, 14–21, and 21+ drinks/day), and serology of hepatitis B and C virus. In subgroup analyses, the stratifying variable was not input as a covariate.

§ Positive for hepatitis B surface antigen, antibodies to hepatitis B core antigen, or antibodies to hepatitis C virus.

¶ Subjects who quit smoking 10 or more years ago were long-term ex-smokers whereas those who quit smoking less than 10 years ago were recent ex-smokers.

We also examined whether the *EGF –* HCC risk association differed across different risk profiles as determined by environmental exposures (use of tobacco or alcohol, viral hepatitis serologic status) or the Th1/Th2 genotypes. Among non-Asians in Los Angeles who consumed 3 or more drinks of alcoholic beverages per day, the age-sex-race-adjusted OR for HCC associated with the *EGF A/G* or *G/G* genotype was 4.34 (95%CI = 1.37–13.74, *P* = 0.01) compared with the *A/A* genotype (Table 3). Further adjustment for additional HCC risk factors slightly diminished the OR to 3.44 (95% CI = 0.93–12.76, *P* = 0.065). Cigarette smoking or chronic infection with HBV and/or HCV did not modify the association between *EGF* genotype and HCC risk. All corresponding associations between *EGF* genotype and HCC risk were null among the Chinese in Guangxi (Table 3).

Table 4 shows the association between *EGF* genotype and risk of HCC in low-risk non-Asians and high-risk Chinese, respectively, stratified by Th1 and/or Th2 genotypes. Among non-Asians with 2 or more low-activity Th1 genotypes, the *EGF A/G* or *G/G* genotypes was associated with a borderline statistically significant increased risk of HCC (OR = 2.77, 95% CI = 1.00–7.66, *P* = 0.049) (Table 4). The corresponding OR among subjects with 1–2 low-activity Th2 genotypes was statistically significant at 2.27 (95% CI = 1.01–5.11, *P* = 0.047). OR was further increased to 3.34 (95% CI = 1.24–9.03, P = 0.017) for individuals possessing the highest-risk Th1/Th2 combined genotype profile. No such modifying effects of Th1 and/or Th2 genotypes on HCC risk were noted among Chinese in southern Guangxi, China.

**Table 4:** **Comparison of *EGF *genotype frequencies in HCC patients with control subjects among non-Asians in Los Angeles, California, and Chinese in southern Guangxi, China, stratified by cytokine genotypes**

Th1 and Th2 genotypes*	***EGF 61A > G***	**Non-Asians in Los Angeles**			**Chinese in southern Guangxi**		
		Ca/Co*	**OR (95% CI)†**	**OR (95% CI)‡**	Ca/Co*	**OR (95% CI)†**	**OR (95% CI)‡**
**No. of low-activity Th1 genotypes**							
0–1	*A/A*	13/27	1.00 (ref)	1.00 (ref)	70/76	1.00 (ref)	1.00 (ref)
	*A/G + G/G*	38/86	1.14 (0.50–2.63)	1.24 (0.46–3.34)	61/77	0.87 (0.54–1.39)	0.63 (0.32–1.23)
2–3	*A/A*	11/25	1.00 (ref)	1.00 (ref)	55/42	1.00 (ref)	1.00 (ref)
	*A/G + G/G*	50/60	2.17 (0.91–5.18)	2.77 (1.00–7.66)	63/50	0.95 (0.55–1.65)	0.89 (0.40v1.97)
**No. of low-activity Th2 genotypes**							
0	*A/A*	7/13	1.00 (ref)	1.00 (ref)	72/61	1.00 (ref)	1.00 (ref)
	*A/G + G/G*	18/28	1.85 (0.48–7.15)	1.36 (0.23–8.27)	55/48	0.97 (0.58–1.63)	0.78 (0.36–1.69)
1–2	*A/A*	17/39	1.00 (ref)	1.00 (ref)	53/57	1.00 (ref)	1.00 (ref)
	*A/G + G/G*	70/118	1.63 (0.83–3.20)	2.27 (1.01–5.11)	69/79	0.95 (0.58–1.56)	0.84 (0.42–1.71)
**No. of low-activity Th1 and Th2 genotypes**							
Th1 = 0–1 and Th2 = 1–2	*A/A*	13/19	1.00 (ref)	1.00 (ref)	35/31	1.00 (ref)	1.00 (ref)
	*A/G + G/G*	33/70	0.86 (0.36–2.04)	0.95 (0.33–2.70)	36/48	0.69 (0.36–1.33)	0.57 (0.21–1.54)
Th1 = 2–3 or Th2 = 0	*A/A*	11/33	1.00 (ref)	1.00 (ref)	90/87	1.00 (ref)	1.00 (ref)
	*A/G + G/G*	55/76	2.75 (1.18–6.38)	3.34 (1.24–9.03)	88/79	1.07 (0.70–1.63)	0.85 (0.47–1.56)

* Number of cases/number of controls; subjects with unknown Th1 and/or Th2 genotypes were excluded from this analysis.

† Adjusted for age, sex, and race; OR, odds ratio; CI, confidence interval.

‡ Further adjusted for level of education, smoking (non-smokers or long-term ex-smokers, current smokers or recent ex-smokers with <20 cigarettes/day, current smokers or recent ex-smokers with 20+ cigarettes/day), alcohol consumption (non-drinkers, <7, 7–14, 14–21, and 21+ drinks/day), and serology of hepatitis B and C virus, if applicable.

## Discussion

EGF induces hepatocyte proliferation in response to liver injury, thereby facilitating liver regeneration [10]. Its role in hepatocellular transformation has been studied both *in vitro* and *in vivo*, showing that EGF enhanced hepatocyte transformation and that EGF over-expression in the liver caused HCC [12,13]. A functional polymorphism at position 61 has been described with *G/G* or *A/G* genotypes associated with significantly higher EGF production both in normal peripheral-blood mononuclear cell cultures [14] and in serum and liver tissues of HCC patients [15]. To our knowledge, population-based, epidemiologic data examining the role of this polymorphism in HCC development were non-existent. The present study shows a positive association between *EGF 61A > G* polymorphism and risk of HCC among low-risk non-Asians in Los Angeles, California, especially among those who were heavy users of alcohol or who possessed a genetic Th1/Th2 profile linked to high HCC risk [22]. In contrast, no association between *EGF* genotype and HCC risk was noted among the native population of Southern Guangxi, China, who exhibit one of the highest incidence of HCC in the world.

The difference in the *EGF-*HCC risk association between the Chinese in southern Guangxi and non-Asians in Los Angeles could be due to the different allele frequencies of *EGF 61A > G* polymorphism between the two study populations. Compared with non-Hispanic whites in Los Angeles, Chinese in Guangxi had a significantly lower frequency of the 61*G allele of the *EGF* gene (55% versus 30%).

We also conjecture that the huge difference in risk of HCC between the Chinese in Southern Guangxi (120/100,000 person-years in men) and the non-Asians of Los Angeles (4/100,000 person-years in men) may be one explanation for the seemingly disparate findings in the *EGF*-HCC association between the two populations. If the EGF genotype effect on HCC occurrence is independent of the combined effect from other risk factors, then the statistical power to detect a given EGF-HCC association should not be influenced by the population’s background level of HCC risk. In other words, a multiplicative model of interaction between *EGF* genotype and background risk factors means one would observe the same magnitude of EGF-HCC association between low-risk non-Asians and high-risk native Chinese whose background level of risk vary by a factor of 30 (120/100,000 versus 4/100,000). But suppose the interaction model is additive rather than multiplicative, then a two-fold risk between low- versus high-risk EGF profile in non-Asians (the result reported here) would translate to a relative risk of 1.07 (32/30) in Chinese of Southern Guangxi. In other words, an additive model of interaction between *EGF* genotype and background risk factors would predict a null finding among the high-risk Chinese.

The observed modifying effect of Th1/Th2 genotypes on the association between EGF and HCC is not surprising, given the well-established interplay of NF-κB and JAK-STAT pathways and cytokine/growth factor signaling in liver regeneration. It’s been proposed that cytokines (predominantly TNFα)-mediated NF-κB activation causes “priming” of hepatocytes to enhance their sensitivity to direct mitogens (such as EGF) in the process of liver regeneration [25]. In addition, growth factors (including EGF) can act as alternative “rescue” activators of NF-κB in the absence of main cytokine signaling [26]. Similarly, STAT3, an important member of the JAK-STAT pathway, is activated by both EGF and cytokines (IL-10, IL-6), and has been proposed to play a central role in viral-induced HCC [27], where IL-6 and EGF were shown to act in concert to promote expression of HBV viral genes [28]. Thus, utilization of common signaling pathways (NF-κB, STAT3) by inflammatory and growth factors provides a framework for their collaboration in liver carcinogenesis [27].

The present study also suggests a role of the *EGF 61A > G* polymorphism in the development of HCC among low-risk non-Asians of Los Angeles who were heavy users of alcohol. EGF can modulate the effect of ethanol on cell proliferation and DNA synthesis [27,29]. A recent study demonstrated that EGF-like growth factors can reduce apoptosis and enhance cell proliferation caused by exposure to alcohol [30]. Given that reduced apoptosis and enhanced cell proliferation are hallmarks of carcinogenesis, these experimental results suggest a plausible biological mechanism for the modifying effect of the *EGF* genetic polymorphism on HCC development among heavy drinkers.

Heavy alcohol consumption is an important risk factor for HCC in non-Asians in Los Angeles. Forty-six percent (n = 54) of HCC patients and 18% of control subjects consumed 3 or more drinks per day among non-Asians in Los Angeles. Of the 54 HCC patients consuming 3 or more alcoholic beverages per day, 22 (41%) were free of HBV or HCV serological markers, two primary risk factors for HCC, indicating that heavy alcohol consumption plays an important role in the HCC development in low-risk non-Asians in Los Angeles, California. In contrast, only 13% (n = 32) Chinese HCC patients and 4% Chinese controls in Guangxi consumed 3 or more drinks of alcoholic beverages per day. Among the 32 HCC patients who consumed 3 or more alcohol drinks per day, 26 (81%) also tested positive for HBsAg and/or anti-HCV. In addition, dietary exposure to aflatoxin has been identified as another major risk factor for HCC in this high-risk population [20]. Therefore, heavy alcohol intake per se plays a relatively minor role in contributing to the burden of HCC in this Chinese population.

The association between *EGF 61A > G* and the risk of developing HCC was initially reported in two independent cohorts of cirrhotic patients; cirrhotic patients possessing the *EGF 61 G/G* genotype had 2- to 4-fold increased risk of HCC compared with cirrhotic patients with the *EGF 61A/A* genotype [15]. These initial findings were replicated in the Hepatitis C Antiviral Long-term Treatment against Cirrhosis (HALT-C) trial cohort; hepatitis C patients with an Ishak fibrosis stage of 3 or higher and possessing the *EGF 61 G/G* genotype experienced a doubling HCC risk relative to their counterparts with the *EGF 61A/A* genotype [31]. Although these studies provided a strong support for a role of *EGF* genetic polymorphism in the progression of liver disease from fibrosis/cirrhosis to malignant stage, these data provide no information on the relationship between *EGF* genetic polymorphism and risk of HCC in a general population as discussed by Galmozzi and Colombo [32]. It should be noted that the average *EGF G* allele frequency among the HCC patients of the two studies described above was 58% [15,31], which was virtually identical to the percentage noted in white HCC patients in the present study (57%).

There were two recent reports of meta-analysis on the association between the *EGF 61A > G* genetic polymorphisms and risk of HCC in Caucasians, Chinese or mixed races [33,34]. The latter meta-analysis [34] was based on data from 8 case–control studies totaling 1304 HCC cases and 2613 controls, which included all six case–control studies examined in the first meta-analysis report [33]. This latter meta-analysis yielded a summary OR of 1.79 (95% CI = 1.39–2.29) for the *EGF 61 G/G* versus the *A/A* genotype [34]. Significantly, this positive association between *EGF 61A > G* polymorphism and HCC was seen only hospital-based studies utilizing hospital controls. No association was observed among studies utilizing population-based controls. In other words, the overall null association noted in the present study is consistent with published literature.

There are inherent limitations in the present study that have been previously described [22,23]. Briefly, both studies were of relatively small sample size. Thus, we were unable to conduct separate analyses for non-Hispanic white Americans and Hispanics/blacks, in spite of their differences in allele frequencies of *EGF 61A > G* polymorphism. Although race was one of the matching factors for the original study design, the imbalance in the race/ethnic distribution between HCC patients and controls of the Los Angeles study was the results of a greater proportion of black and Hispanic controls who refused to donate blood samples [6]. In addition, the majority of eligible patients died before we were able to approach them for study participation due to the rapidly fatal nature of HCC following clinical diagnosis. Nonetheless, demographic features of eligible patients who were excluded from the study were similar to those who participated in our study. For the study in Guangxi, China, we were unable to review medical charts or obtain liver tissue slides to confirm the diagnosis of HCC and to estimate the prevalence of cirrhosis among HCC patients. If cirrhosis might modulate the influence of *EGF* polymorphism on HCC risk, the difference in the underlying etiologies for HCC between Chinese population in China (e.g., hepatitis B and dietary aflatoxin exposure) and non-Asians in Los Angeles (e.g., hepatitis C and alcohol abuse) could explain partially the observed differences in the *EGF*-HCC risk association between the two study populations since some of HCC patients with hepatitis B as the underlying cause did not show cirrhosis in the liver.

## Conclusions

The polymorphism in *EGF* gene associated with its increased expression was linked to HCC development in a low-risk non-Asian population, while no such association was observed in a high-risk Chinese population. Among the low-risk non-Asians, the *EGF* gene-HCC risk associations were confined to heavy alcohol drinkers and subjects possessing the high-risk Th1/Th2 genotypic profiles.

## Abbreviations

Anti-HBc: Antibodies to the hepatitis B core antigen; Anti-HCV: Antibodies to the hepatitis C virus; CI: Confidence interval; EGF: Epidermal growth factor; HBsAg: Hepatitis B surface antigen; HBV: Hepatitis B virus; HCC: Hepatocellular carcinoma; HCV: Hepatitis C virus; OR: Odds ratio; Th1: T-helper 1; Th2: T-helper 2.

## Competing interests

The authors declare that they have no competing interests.

## Authors’ contributions

Dr. Yuan had full access to all of the data in the study and takes responsibility for the integrity of the data and the accuracy of the data analysis. Study concept and design: JMY, MCY. Acquisition of data: JMY, DVDB, SG, MCY. Analysis and interpretation of data: JMY, YF, RW, MCY. Drafting of the manuscript: JMY, YF, SO. Critical revision of the manuscript for important intellectual content: JMY, MCY. Statistical analysis: JMY, YF, RW. Obtaining funding: JMY, MCY. Administrative, technical, or material support: JMY, YF, RW, DVDB, SG, MCY. All authors read and approved the final manuscript.

## Pre-publication history

The pre-publication history for this paper can be accessed here:

http://www.biomedcentral.com/1471-230X/13/32/prepub
